# Genetic assessment reveals no population substructure and divergent regional and sex-specific histories in the Chachapoyas from northeast Peru

**DOI:** 10.1371/journal.pone.0244497

**Published:** 2020-12-31

**Authors:** Evelyn K. Guevara, Jukka U. Palo, Sanni Översti, Jonathan L. King, Maria Seidel, Monika Stoljarova, Frank R. Wendt, Magdalena M. Bus, Anna Guengerich, Warren B. Church, Sonia Guillén, Lutz Roewer, Bruce Budowle, Antti Sajantila

**Affiliations:** 1 Department of Forensic Medicine, University of Helsinki, Helsinki, Finland; 2 Forensic Genetics Unit, Finnish Institute for Health and Welfare, Helsinki, Finland; 3 Department of Biosciences, University of Helsinki, Helsinki, Finland; 4 Center for Human Identification, University of North Texas Health Science Center, Fort Worth, Texas, United States of America; 5 Department of Forensic Genetics, Institute of Legal Medicine and Forensic Sciences, Charité – Universitätsmedizin Berlin, Berlin, Germany; 6 Department of Chemistry and Biotechnology, Tallinn University of Technology, Tallinn, Estonia; 7 Department of Psychiatry, Yale University School of Medicine and VA Connecticut Healthcare System, West Haven, Connecticut, United States of America; 8 Department of Microbiology, Immunology and Genetics, Graduate School of Biomedical Sciences, University of North Texas Health Science Center, Fort Worth, Texas, United States of America; 9 Eckerd College, Saint Petersburg, Florida, United States of America; 10 Department of Earth and Space Sciences, Columbus State University, Columbus, Georgia, United States of America; 11 Centro Mallqui, San Isidro, Lima, Peru; 12 Forensic Medicine Unit, Finnish Institute for Health and Welfare, Helsinki, Finland; Illumina Inc, UNITED STATES

## Abstract

Many native populations in South America have been severely impacted by two relatively recent historical events, the Inca and the Spanish conquest. However decisive these disruptive events may have been, the populations and their gene pools have been shaped markedly also by the history prior to the conquests. This study focuses mainly on the Chachapoya peoples that inhabit the montane forests on the eastern slopes of the northern Peruvian Andes, but also includes three distinct neighboring populations (the Jívaro, the Huancas and the Cajamarca). By assessing mitochondrial, Y-chromosomal and autosomal diversity in the region, we explore questions that have emerged from archaeological and historical studies of the regional culture (s). These studies have shown, among others, that Chachapoyas was a crossroads for Coast-Andes-Amazon interactions since very early times. In this study, we examine the following questions: 1) was there pre-Hispanic genetic population substructure in the Chachapoyas sample? 2) did the Spanish conquest cause a more severe population decline on Chachapoyan males than on females? 3) can we detect different patterns of European gene flow in the Chachapoyas region? and, 4) did the demographic history in the Chachapoyas resemble the one from the Andean area? Despite cultural differences within the Chachapoyas region as shown by archaeological and ethnohistorical research, genetic markers show no significant evidence for past or current population substructure, although an Amazonian gene flow dynamic in the northern part of this territory is suggested. The data also indicates a bottleneck c. 25 generations ago that was more severe among males than females, as well as divergent population histories for populations in the Andean and Amazonian regions. In line with previous studies, we observe high genetic diversity in the Chachapoyas, despite the documented dramatic population declines. The diverse topography and great biodiversity of the northeastern Peruvian montane forests are potential contributing agents in shaping and maintaining the high genetic diversity in the Chachapoyas region.

## Introduction

Chachapoya culture developed in a singular and biodiverse zone between the Andean mountain range and the Amazonian rainforests from northeastern Peru, and fluoresced around 900 CE (Common Era). Populations thrived in the area until conquered by the Incas (around 1475 CE) and Spaniards (first half of 16^th^ Century). Since the Chachapoyas region has been an important area even before the Late intermediate Period (1000–1475 CE), it has been a target of various archaeological, ethnohistoric, and genetic studies. Previous genetic assessments have demonstrated that the Chachapoyas region today harbors, unlike many other regions inhabited by Native South Americans, high levels of genetic diversity [1, 2], and they also have succeeded in placing Chachapoya populations on the genetic map of South America. However, several questions arising from the fields of archaeology and ethnohistory, remain to be explored with genetic data. These focus on the period of Inca and Spanish conquests in the 15^th^ and 16^th^ century and include a long-standing question regarding population substructure within Chachapoyas, as well as the sex-specific demographic impacts of the conquests. At a somewhat broader scale, we consider the issue of Chachapoyan affinities to human groups in the highland Andes and in lowland Amazonia.

Internal population substructure is of interest as the Chachapoya most likely have not been a homogeneous culture or single ethnic group. In fact, the name “Chachapoyas” was originally given by the Incas to denote an administrational province that held an aggregate of ethnic groups, and the name was later retained by the Spanish [3–5]. Similarly, in archaeology, Chachapoyas is still used as a shorthand to refer collectively to the several culturally diverse groups that specialists distinguish as inhabitants of this region before the Inca and Spanish conquests [6]. It is however unclear, whether the cultural differences identified also signify restricted gene flow and genetic segregation of these subgroups.

As in many other parts of South America, the Incan and European conquests had a profound demographic impact on the human groups in the Chachapoyas region, especially on males, who were particularly recruited for state-labor as well as warfare [4, 6, 7]. Around the years 1450–1475 CE, the Chachapoya territory was annexed as a province within the vast Inca realm, called Tawantinsuyo. Ethnohistorical studies indicate that after the Inca conquest, Chachapoya people were transferred to 18 different locations across Tawantinsuyo [4, 8, 9] and various groups of migrants were relocated into the Chachapoyas province from areas including Cajamarca, the North Coast, and Xauxa [4]. During the Inca civil war thousands of Chachapoya troops were recruited by Huascar to fight against Atahualpa [4]. Later, during the early colonial period, Chachapoyan males were also conscripted into the Spanish militia to fight against the Incas [10] and to explore unconquered Amazonian rainforest territories [4, 10]. European settlers also introduced pathogens that caused a series of recurrent epidemics that decimated the indigenous populations across Peru [11]. In Chachapoyas region, this population decline is reported as dramatic and comparable to the ones experienced in coastal cities, the ports of entry of European migrants [6, 11].

Following the Spanish conquest, the introduction of European genes created an asymmetric genomic landscape, with male-mediated gene flow [1] but also different regions within Chachapoyas were likely being differently impacted by this process. In light of this history, the high native genetic diversity in the Chachapoya region reported today [1, 2] is intriguing.

Here we address the demographic impact in view of the population history outlined above, using autosomal, mitochondrial and Y-chromosomal marker data produced for four northeastern Peruvian groups: Chachapoya, Jívaro, Huancas and Cajamarca. We examine the following questions: 1) was there pre-Hispanic genetic population substructure in the Chachapoyas sample? 2) did the Spanish conquest cause a more severe population decline on Chachapoyan males than on females? 3) can we detect different patterns of European gene flow in the Chachapoyas region? and 4) did the demographic history in the Chachapoyas resemble the one from the Andean area?

## Materials and methods

**Fig 1:**
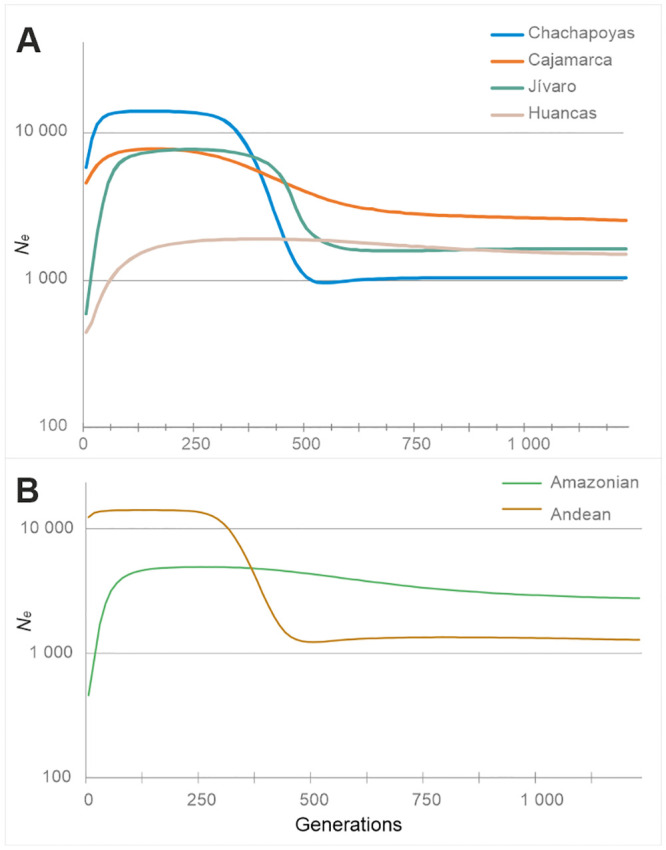
Coding region BSPs. (A) Study populations. (B) Macro-regions Andean (*N* = 72) and Amazonian (*N* = 52).

This study followed ethical guidelines and standards (Helsinki declaration) and was approved by Helsinki University Hospital Ethical Committee (Decision #329/13/03/00/13) as previously described in [1]. Local board permits from Peru were previously obtained (Decision letters: N° 140-2009-GRAMAZONAS-PR and N° 04-2010/GRAMAZONAS-PR). Written informed consents were obtained from all participants. Altogether 246 individuals from three regions were included in this study: 1) the historical area identified as Chachapoyas, including the enclave population from the town of Huancas, 2) two Jivaroan (Chicham) populations (Awajún and Wampís) from the Amazonian rainforest, and 3) individuals from the neighboring Andean region of Cajamarca (The location of study populations can be found on Fig 1 of reference #1). These samples were genotyped by sequencing entire mitochondrial genomes (*N* = 172), Y-chromosomal (*N* = 190) and autosomal markers (*N* = 246).

For the analyses of substructure within Chachapoyas, the samples were divided into seven subgroups: Pomacochas, Chillao, Corobamba, Rodríguez de Mendoza, Chachapoya, La Jalca and Leymebamba. The assignment of individuals into a given subgroup was based on the place of birth up to the grandparents’ generation and followed the strategy outlined in [1]. Briefly, individuals from nearby towns and villages were pooled into the same subgroup if there was documented historical affiliation (colonial and/or modern sources) or if they were united by archaeologically distinct patterns of material culture.

### Genotyping

DNA extractions and Y chromosome short tandem repeat (STR) genotyping have been described in [1]. Full mitochondrial genomes were sequenced following a protocol described in [12]. The VCF files obtained from the Illumina MiSeq FGx system were converted into haplotypes using MitoSAVE [13]. Mitochondrial variants were curated manually and verified independently by three authors (EKG, FW and MS) using Integrative Genomic Viewer (IGV) software [14] Poly-C stretches at positions 303–315 and 16184–16193 were excluded from subsequent analyses. Sequences were aligned to the revised Cambridge Reference Sequence (rCRS) [15] using CLUSTAL Omega [16, 17].

For the Y single nucleotide polymorphisms (Y-SNPs) analyses eight multiplex assays were used: MI and MII [18]; SpecE, SpecI and SpecJ [19]; SpecR [20]; and SpecQ-M346 and SpecQ-M3 (S1 Table)

Autosomal genotyping was carried out using the ForenSeq^™^ DNA Signature Prep Kit (Verogen; San Diego, Ca., USA) as described elsewhere [21]. Output FASTQ files from the ForenSeq^™^ Universal Analysis Software (UAS) pipeline were processed with STRait Razor v2s [22] to obtain the genotypes.

After data curation and filtering, 164 mitochondrial genomes (GenBank: MN857248–MN857411), 177 Y-chromosome (new Y-SNPs data) and 241 autosomal (27 short tandem repeats, STRs, and 94 single nucleotide polymorphisms, SNPs) profiles from four Peruvian populations (Chachapoyas, Huancas, Jívaro and Cajamarca) were used in downstream analyses.

### Data analysis

#### Mitochondrial genomes

*Haplogroup composition*, *basic statistics and genetic distances*. For the analyses, the newly produced mitochondrial genomes were merged with data from [2]. Additionally, data from 78 worldwide reference populations were included (S2 Table).

Haplogroup assignments were obtained from HaploGrep v2.2 based on Phylotree 17 [23, 24]. Haplotypes belonging to non-Native American haplogroups H and U were excluded from the analyses addressing pre-contact population substructure and past effective population size. The effect of sample sizes on observed haplogroup frequencies was tested by permutations. Here, the number of each macro haplogroup observed was contrasted against the distribution obtained by 100,000 draws of *N* haplotypes, with *N* corresponding to the sample size in each subpopulation. In the permutations, the success of drawing a certain haplotype was determined by its frequency in the modern Peruvian reference population (*N* = 442).

Summary statistics, e.g. haplotype diversity (*h*), nucleotide diversity (*π*), mean number of pairwise differences (*MNPD*), and pairwise genetic distances (*Φ*_*ST*_) were estimated using Arlequin ver. 3.11 [25]. Genetic distances between populations were visualized with multi-dimensional scaling (MDS) plots built in RStudio version 3.5.3 with the package *cmdscale*.

*Bayesian Skyline Plots (BSPs)*. Maternal demographic histories were explored with BSPs constructed using mitochondrial coding region data (nucleotides 577–16023) at three levels: 1) local (Chachapoyan subgroups), 2) regional (Chachapoyas, Huancas, Jivaro, Cajamarca), 3) subcontinental (Andean and Amazonian macro-regions). The best-fit substitution model for each population was determined with bModelTest [26]; the substitution model used for each population are described in S3 Table. BSPs were constructed using BEAST 2 v.2.4.7 [27] assuming discrete gamma distribution with four categories. As a tree prior, the piecewise-linear Skyline coalescent model with ten groups was used. The prior normal distribution for mutation rate was estimated based on six previously published mutation rates for mitochondrial coding region [28–33]. The mean mutation rate used was *μ* = 1.546 x 10^−8^ ± 3.675 x 10^−9^ (SD) substitutions/pair/year. For each population, two molecular clocks were tested: strict and log normal relaxed molecular clock and the best-fit molecular clock was determined with Akaike information criterion method (AICM) implemented in Tracer [34]. The resulting clock models used for each population are presented in S3 Table. For each BEAST run, the Markov chain Monte Carlo (MCMC) length was set to 10,000,000 steps, with the first 10% of the steps discarded as burn-in and the chain sampled every 1,000 steps. Three independent runs were performed and combined with LogCombiner (part of BEAST package). Adequate effective sample sizes (ESS > 200) were checked in Tracer v.1.7.0 [35] for each of the parameters.

#### Y-STRs and Y-SNPs

*Haplogroup composition*, *basic statistics and genetic distances*. Similar to the mitochondrial DNA data, our Chachapoya Y-chromosomal dataset was merged with the data from [2]. In addition, STR data from 88 worldwide (23 STRs) and 79 Native American (17 STRs) reference populations were included (S2 Table). The Y-haplogroups were determined based on the Y-SNP data and also estimated based on the Y-STR haplotypes using a Haplogroup Predictor (http://www.hprg.com/hapest5/). Summary statistics were calculated in a similar fashion as the mitochondrial data using Arlequin ver.3.11.

*Bayesian skyline plots*. Demographic histories were assessed by constructing BSPs for the Chachapoyas pooled, as well as for one Andean and one Amazonian set. The male demographic history was explored by reconstructing Bayesian skyline plots with BEASTvntr package [36] implemented in BEAST v2.4.7. The Sainudiin model [37] with computed frequencies was used, together with the uncorrelated relaxed lognormal clock model. Sample sizes, other prior distributions and parameters are displayed in S4 Table. Tree calibration was accomplished by setting the prior distribution for mutation and the node calibration, and by assuming an evolutionary mutation rate of 8.2 x 10^−4^ ± 5.7 x 10^−4^ mutations/locus/generation calculated based on loci specific estimates presented in [38]. A node calibration approach was also used. However, as all Peruvian samples included belong to haplogroup Q its node calibration cannot be used directly. For this reason, an outgroup of six individuals from haplogroup R (sub-haplogroups R1a1a, R1a2, R1b11, R1b3, R2a and R2; extracted from [39]) were included in the analysis and the age estimate for a common node P1 was fixed. The P1 nodal age was set according to the divergence date determined based on the Y-chromosomal sequence data [39]. We assumed a normal distribution with mean = 1411 generations and SD = 100. This covered the P1 age obtained in [39] (mean = 35282 years before present (ybp) and 95% highest posterior density [33662, 36917] ybp), assuming a male generation time of 25 years. The settings for each BEAST run, the number of runs, and downstream procedures with LogCombiner and Tracer v.1.7.0, were identical to the ones used for mitochondrial data.

#### Autosomal STR and SNP data

In case of the autosomal data, the Chachapoyan samples were allocated to subgroups in a slightly different manner than with the lineage markers. The Kuelap in Chachapoya, Uchucmarca in Leymebamba and the original Jivaroan sample collection groups Awajún and Wampís were analyzed as separate units. Additionally, 31 reference populations were used (S2 Table). For five of these populations 121 markers (27 autosomal STRs and 94 SNPs) were available and for the remaining populations less than 19 autosomal STRs. Allele frequencies were calculated and tested for statistically significant departures (*p* < 0.05) from Hardy-Weinberg Equilibrium (HWE) and Linkage disequilibrium (LD) using Arlequin ver. 3.11.

*Genetic distances and population structure*. Pairwise genetic distances (*F*_ST_) between populations were calculated in Arlequin ver. 3.11. Genetic substructure and admixture proportions were calculated at three different hierarchical levels: 1) locally within Chachapoyas, 2) intra-continentally (Americas) and 3) inter-continentally, using STRUCTURE v 2.3.4 [40–43]. For the intracontinental analysis, published reference data from 15 populations from the Americas were incorporated. Due to the limited availability of reference data, the analyses were based on 11 and 15 aSTR loci. For the intercontinental analysis, reference populations from African Americans (AFA), European descendants in the USA (CAU), Asian Americans (ASN) and Hispanics (HIS) from [44] were included. Downstream analyses used 27 autosomal STRs and 94 autosomal SNPs.

For the STRUCTURE analyses, the parameter lambda (*λ*) was estimated for our whole dataset and the resulting value (*λ* = 0.5) was fixed in all runs. The parameter *alpha* (α) was estimated independently for each population with an initial value of one. The admixture model chosen was correlated alleles and 8–12 iterations were calculated in each run. Convergence in the hyperparameters such as *alpha* and *F* was observed after c. 60,000 steps in both burn-in and run length. Number of ancestry components, *K*, was chosen based on two criteria, the estimated log probability of the data (*lnP* (*X*|*K*) [40] and delta K (Δ*K*) [45]. Cluster permutations and plotting of successive values of *K* from 1 to 7 were carried out with CLUMPAK main pipeline [46]. An alternative method to explore population substructure was Principal Coordinates Analyses (PCoA), for which Genalex add-in for Excel [47] and PAST 3.24 [48] were used. In addition to these methods, Analysis of Molecular Variance (AMOVA) for all marker systems was also performed in Arlequin ver. 3.11 [25].

## Results

### Mitochondrial DNA

#### Haplogroup composition

Among the newly produced data three mitochondrial lineages previously unreported in Phylotree17 were observed. Roughly 97% of the samples belonged to Native American haplogroups A2, B2, C1 and D (Table 1). The non-Native American haplogroups H and U were only observed in the Chachapoyas set with a frequency of 4.9%. The most frequent haplogroup is B2 (43.9%), followed by haplogroups C1 (24.4%), D (16.5%) and A2 (12.2%). In the whole mitochondrial dataset, larger variability of sub-haplogroups is observed within haplogroups A2 and B2, the former being the most diverse with five different sub-clades (S5 Table).

**Table 1:** Absolute and relative (%) mitochondrial haplogroup frequencies for all study populations*.

Population/Haplogroup	*N*	Native American				European	
		A2	B2	C1	D	H	U
Chachapoya	20	5 (25)	6 (30)	3 (15)	5 (25)	1 (5)	0
Chillao	10	3 (30)	3 (30)	3 (30)	0	1(10)	0
Corobamba	9	1 (11.1)	2 (22.2)	4 (44.4)	2 (22.2)	0	0
La Jalca	9	1 (11.1)	5 (55.5)	2 (22.2)	1 (11.1)	0	0
Leymebamba	14	1 (7.1)	4 (28.6)	4 (28.6)	5 (35.7)	0	0
Pomacochas	13	1 (7.7)	10 (76.9)	1 (7.7)	1 (7.7)	0	0
Rodríguez de Mendoza	27	0	8 (29.6)	8 (29.6)	8 (29.6)	2 (7.4)	1 (3.7)
**Chachapoyas pooled**	**102**	12 (11.8)	38 (37.3)	25 (24.5)	22 (21.6)	4 (3.9)	1 (1)
**Jívaro**	**42**	5 (11.9)	27 (64.3)	9 (21.4)	1 (2.4)	0	0
**Huancas**	**4**	1 (25.0)	2 (50)	0	1 (25)	0	0
**Cajamarca**	**16**	2 (12.5)	5 (31.3)	6 (37.5)	3 (18.8)	0	0
**ALL COMBINED**	**164**	20 (12.2)	72 (43.9)	40 (24.4)	27 (16.5)	4 (2.4)	1 (0.6)

* Figures only from our datasets.

Among the seven Chachapoyan subgroups, differences in haplogroup distribution were apparent, e.g. Chillao lacks haplogroup D, whereas the Rodriguez de Mendoza lacks haplogroup A2 (Table 1). In order to assess whether these observations were the result of chance alone, random sampling procedures were simulated for each study population and in each Chachapoyan subgroup. In case of most haplogroups, the number of observations fall inside a simulated distribution assuming average Peruvian haplogroup frequencies. However, both in Jívaro and Pomacochas, haplogroup B2 was significantly more common than expected, as was haplogroup H in Chillao and Rodriquez de Mendoza. Rodriquez de Mendoza lacked haplogroup A2 completely, when one would expect 2–9 observations in a sample of 27 individuals, and haplogroup D was underrepresented in the Jivaroan sample (S1 Fig).

#### Summary statistics and genetic distances

Considering the merged datasets (this study and Barbieri et al. 2017), high levels of genetic diversity (e.g. *h*, *π*) were observed in all study populations (S6 Table) even when the non-Native American haplotypes were excluded from the analysis. Genetic distances among the Chachapoyan subgroups revealed that Pomacochas differentiated significantly from all the others (*Φ*_ST_ ≥ 0.1, *p* ≤ 0.05; S7 Table) and Rodríguez de Mendoza differentiated from Chachapoya, Chillao and Pomacochas subgroups. The observed genetic distances increased consistently (by 1–20 percentage points) when haplotypes belonging to non-Native American haplogroups were excluded. Since the sample sizes of all Chachapoyan subgroups are relatively small, real affinities cannot be differentiated from the effects of sampling error. In that sense, only subgroups showing significant differentiation, such as Pomacochas and Rodríguez de Mendoza, were kept independent; while the remaining were merged into a combined set labeled Chachapoyas (S8 Table).

The Chachapoyas pooled had genetic affinities (non-significant) with the Cajamarca but remained distant to the Jívaro and the Huancas. Genetic distances in the context of the Americas are summarized in an MDS plot where the clumped Chachapoyas set is positioned near the centroid. The Pomacochas falls in a group that includes the Amazonian Jívaro and populations from Ecuador. The Rodríguez de Mendoza set situates instead close to ancient Peruvian populations (S2 Fig).

#### Mitochondrial BSPs

The Bayesian Skyline plots based on the mitochondrial coding region are shown in Fig 1 and S3 Fig. The graphs exhibit a marked population growth in the distant past, which starts around 500 generations ago and reaches a plateau c. 250 generations ago. For the pooled Chachapoyas, as well as for all the subgroups, the plots suggest a reduction of *N*_e_ starting at around 125 generations ago (~3 kya) and persisting forward in time. The observed pattern resembles the one observed for the Andean region, but differs markedly from the Amazonian one which do not show prominent growth at any stage in the past (Fig 1B). However, the reduction of *N*_e_ around 3 kya appears as a common feature in most study and reference populations.

### Y-STRs and Y-SNPs

#### Haplogroup composition

Y-STR haplotypes were previously reported in [1]. Considering FTDNA order and equal priors, the new Y-SNP data revealed 12 differences in the haplogroup assignments by the STR haplogroup predictor used (S9 Table). Around 64% of the haplotypes belonged to Native American haplogroup Q followed by non-Native American haplogroup R (19.2%). The Chachapoyas pooled set and the Cajamarca have different proportions of non-Native American haplogroups other than R (Table 2). Locally, within Chachapoyas, haplogroup Q is found in high frequencies in all subgroups with the exception of Rodríguez de Mendoza where haplogroup R is predominant (41.2%). Overall, haplogroups Q, J, and R, each with three lineages, are the most diverse in the whole dataset (S10 Table).

**Table 2:** Absolute and relative (%) Y-chromosome haplogroup frequencies for all study populations*.

Population/Haplogroup	*N*	Native American	Eurasian					African
		Q	G-M201	I-P37.2	J	R	KL-M9	E-M35
Chachapoyas	24	12 (50.0)	1 (4.2)	1 (4.2)	1 (4.2)	8 (33.3)	0	1 (4.2)
Chillao	14	12 (85.7)	0	0	2 (14.3)	0	0	0
Corobamba	7	6 (85.7)	0	0	1 (14.3)	0	0	0
La Jalca	17	13 (76.5)	0	0	0	4 (23.5)	0	0
Leymebamba	19	8 (42.1)	0	1 (5.3)	5 (26.3)	3 (15.8)	0	2 (10.5)
Pomacochas	22	17 (77.3)	2 (9.1)	0	0	2 (9.1)	0	1 (4.5)
Rodríguez de Mendoza	17	2 (11.8)	3 (17.6)	1 (5.9)	3 (17.6)	7 (41.2)	1 (5.9)	0
**Chachapoyas pooled**	120	70 (58.3)	6 (5.0)	3 (2.5)	12 (10.0)	24 (20.0)	1 (0.8)	4 (3.3)
**Jivaro**	24	23 (95.8)	0	0	0	1 (4.2)	0	0
**Huancas**	12	11 (91.7)	0	0	0	1 (8.3)	0	0
**Cajamarca**	21	9 (43)	2 (9.5)	0	2 (9.5)	8 (38)	0	0
**ALL COMBINED**	177	113 (63.8)	8 (4.5)	3 (1.7)	14 (7.9)	34 (19.2)	1 (0.6)	4 (2.3)

*Figures only from our datasets.

#### Summary statistics and genetic distances

Considering the merged datasets (this study and Barbieri et al. 2017), all study populations exhibit high levels of diversity (e.g. *h*, *π*) when using Y-STR markers (S11 Table), which is also consistent when excluding the non-Native American haplotypes. Considering these 23 markers, genetic distances reveal that Pomacochas differentiated (*Φ*_ST_ ≥ 0.1, *p* ≤ 0.05) from most of the other subgroups (S12 Table). After pooling the Chachapoyan subgroups, 17 STRs were used for comparisons in order to include more populations from the Americas (S12 Table). Here the Chachapoyas had affinities (*Φ*_ST_ ≤ 0.05, *p* <0.05) only with the Huancas and remained distant to both the Jívaro and the Cajamarca. The Jivaroan set did not have any affinities with study or reference populations. In the MDS plot, the Chachapoyas pooled set falls in between Andean and Amazonian clusters very close to the Huancas. The subgroups Pomacochas and La Jalca are relatively close to each other but slightly distant to the pooled Chachapoyas. The Jívaro situates in the periphery of the plot, similar to other Amazonian groups, while the Cajamarca falls within an Andean cluster (S4 Fig).

#### Y-chromosomal BSPs

**Fig 2:**
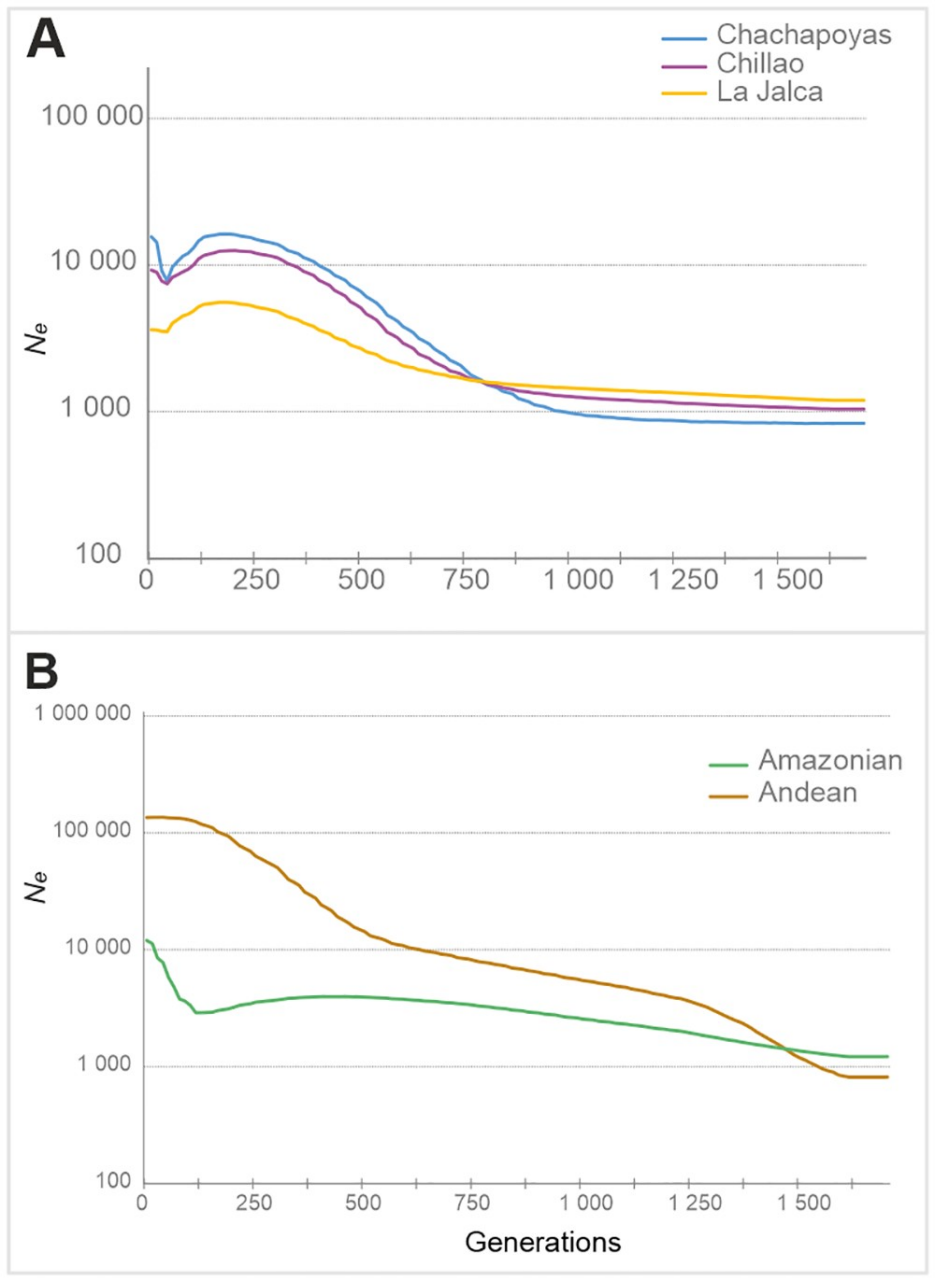
BSP with 21 Y-STRs treated as one partition. (A) For the Chillao, La Jalca and Chachapoyas pooled. (B) Macro-regions Andean (*N* = 81) and Amazonian (*N* = 37).

Considering the full Chachapoyas set and assuming all Y-STRs as one partition with *μ* = 8.2 x 10^−4^ ± 5.7 x 10^−4^ per generation, a marked population growth is observed at around the same time as for the mitochondrial data, from 600–200 generations (15–5 kya, assuming a male generation time of 25 years). Population decline for this set starts at around 200 generations and persists until it reaches its lowest point about 25 generations ago (c. 625 years ago assuming the mutation rate above) (Fig 2A). This deep *N*_*e*_ contraction of about 50% since the beginning of the population decline parallels either the time of Inca or Spanish conquest (22 or 25 years/generation interval, respectively). After this event, there is a continuous increase of the population size of more than 20% since reaching the lowest point, which is absent in the BSPs for mitochondrial data. Among the reference sets, the Amazonian one shows a very modest population increase starting at 800 generations ago (20 kya), which reaches a maximum height at 585 generations ago (~15 kya). After this, the population size declines slowly until reaching its lowest point at 110 generations ago (~2.8 kya). Following this recent population contraction, a rebound in *N*_e_ similar to the one observed in the Chachapoyas is detected for the Amazonian set (Fig 2B). The BSP for the Andean region in turn shows two intervals of population growth. The first one reaching its highest point at 512 generations ago (12.8 kya) and the second one at 73 generations ago (1.8 kya), after which there is no population decline in the recent past.

### Autosomal

#### Summary statistics

All loci were in Hardy-Weinberg equilibrium in all populations with the exception of locus D1S1656 in the Chachapoyas. Linkage disequilibrium (LD), was assessed for 27 aSTRs and 94 aSNPs in each study population. After correction for multiple testing (*p* < 0.0001), two pairs of loci showed departures from expectations, one in the Chachapoyas (rs13182883–rs338882), and one pair in the Cajamarca (rs6955448–rs917118). Genetic distances showed affinities (*F*_ST_ < 0.05, *p* ≤ 0.05) among all study populations and the Hispanic set (HIS). The Caucasian dataset (CAU) in turn exhibited short genetic distances (*F*_ST_ ≤ 0.02, *p* ≤ 0.05) only to the Chachapoyas, the Awajún and the Cajamarca.

#### Population structure inference

**Fig 3:**
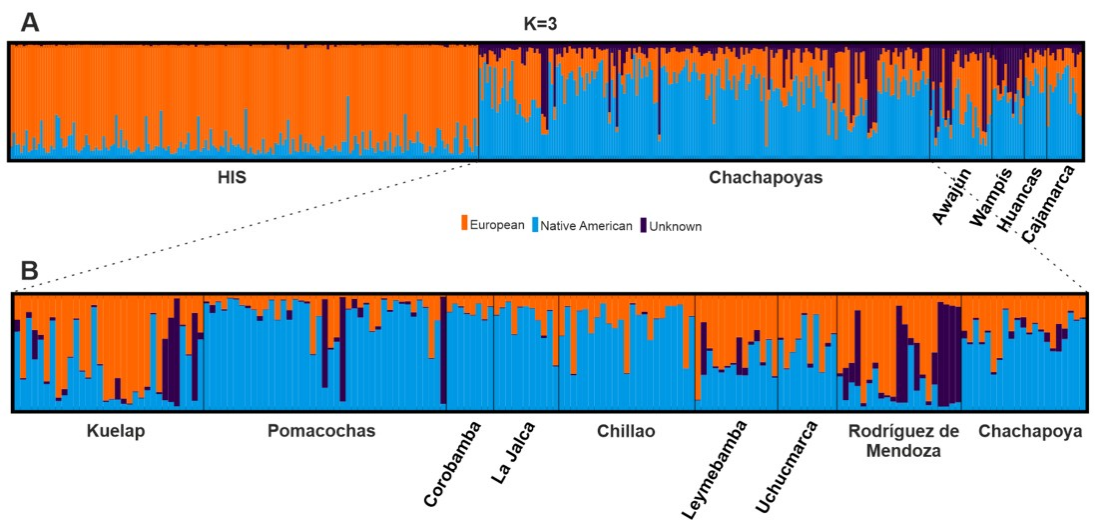
Admixture plots. (A) Study populations and the Hispanic reference (HIS) for *K* = 3. (B) Distribution of the 3^rd^ unknown component within Chachapoyas. This component exhibits higher frequency in the Chachapoyan subgroups Kuelap, Pomacochas and Rodríguez de Mendoza.

Underlying substructure in the study populations was investigated hierarchically. At the upper level, ln *P* (*X*|*K*) and Δ*K* showed four (*K* = 4) well defined clusters represented by Africans (AFA), Europeans (CAU), Asians (ASN) and Native Americans (HIS and study populations) (S5 Fig). When only populations from the Americas were examined, the clustering showed two major components commonly found among most admixed populations from the Americas: European and Native American (Fig 3A). Additionally, the analysis showed a third unknown component present in much higher proportion in three study populations, Chachapoyas, Awajún and Wampís; but nearly absent in the reference Hispanic population (HIS). The 3^rd^ unknown component was consistently observed with increasing number of *K* in plots including also other continental datasets (S1 File). Further examination, only within Chachapoyas (Fig 3B), showed consistently this 3^rd^ component even when the analyses were restricted to a reduced number of markers (18–27 loci, not shown). This finding was supported by both ln *P* (*X*|*K*) and *ΔK* cross validation errors for *K* = 3 (S6 Fig). Zooming into the Chachapoyan subgroups, this third component found is more frequent in Kuelap, Pomacochas and Rodríguez de Mendoza (Fig 3B). The Native American component (light blue) is consistently observed at more than 50% in samples from Chachapoya, Chillao and Pomacochas subgroups. On the other hand, the European component (orange) is often found at higher frequencies (> 50%) in Leymebamba, Rodríguez de Mendoza and Kuelap.

#### PCoA and genetic distances

**Fig 4:**
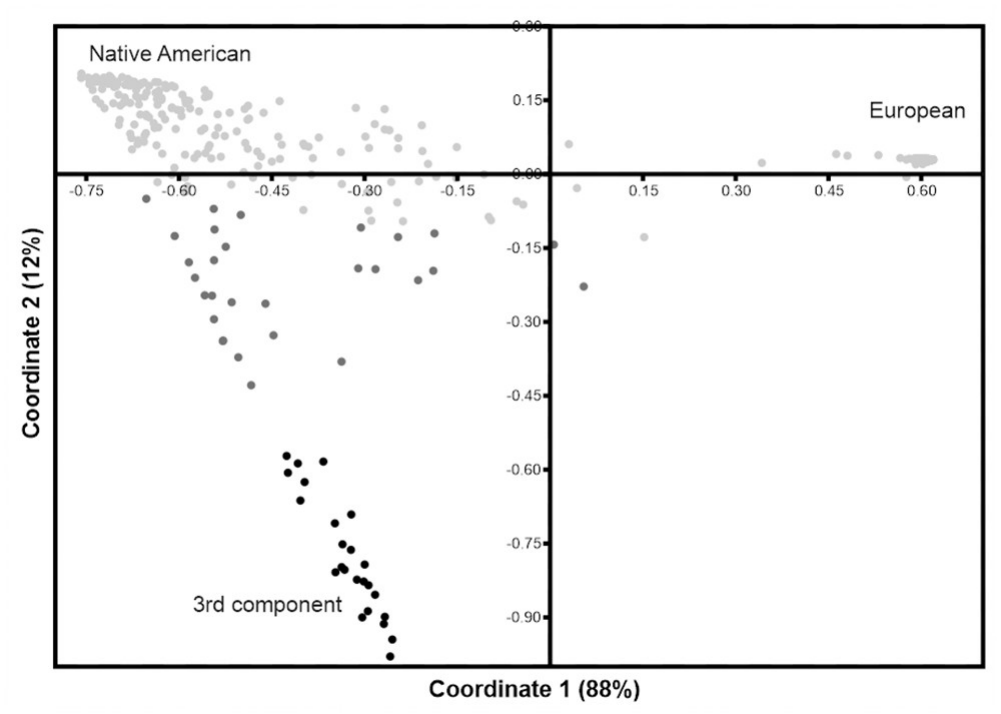
PCoA plot when samples are binned by their proportion of the 3^rd^ unknown component. Individuals harboring more than 60% of this “3^rd^ unknown component” in black.

When performing a PCoA including only the reference set having European ancestry (CAU) and the study populations (S7 Fig), it is possible to identify a small cluster composed of individuals only from three study populations, Chachapoyas, Awajún and Wampís. In this plot, the X-axis separates the European from the Native American clusters while the Y-axis separates the components within the Americas. The STRUCTURE output was used to better visualize the individuals that exhibit more than 60% of the third unidentified component. In order to accomplished this, all samples were binned by the proportion of the third unknown component (purple, in Fig 3), which resulted in three classes: light grey = 0–20%, grey = 21–59% and, black = 60–100% and then plotted in a new PCoA (Fig 4). The separation of European and Native American clusters is similar to the one described for S7 Fig. Individuals that harbor ≥ 60% of the “3^rd^ unknown component” fall close to each other (black dots) in a cline towards the Native American cluster.

To resolve the origins of the “3^rd^ unknown component”, the genotypes of individuals harboring more than 60% of this component were placed in a new synthetic “population” and compared to other populations worldwide. These comparisons were carried out both with a larger set of markers 116 loci (S8A Fig) and with 10 aSTR loci (S8B Fig). With the 10 aSTR set, the “3^rd^ unknown component” only shows affinities (*Φ*_ST_ ≤ 0.05, *p* ≤ 0.05) with the Hispanic reference population from the US (HIS), urban populations from Mexico (Tijuana and Sonora), Chile (Calchaquí Valley) and a Mestizo sample from Nicaragua. On the other hand, the “3^rd^ unknown component” remained distant (*Φ*_*ST*_ ≥ 0.07, *p* ≤ 0.05) to the reference European populations, CAU from the US and Huelva from Spain. In the MDS plot with the full set of markers (116 loci), the “3^rd^ unknown component” remains distant to all study and reference populations. With a reduced set of markers (10 aSTR loci) and more reference populations from Peru, the “3^rd^ unknown component” falls in the periphery of the plot close to the Amazonian Cashibo (S8B Fig).

AMOVA was also used to investigate the distribution of diversity in a number of alternative subpopulation grouping schemes, based on the observed genetic distances between subgroups. As with the clustering analyses (STRUCTURE), AMOVA for autosomal data did not uncover population substructure within Chachapoyas, both *F*_*CT*_ (among groups) and *F*_*SC*_ (among populations within groups) values were non-significant and rather very small (S13 Table, run: 11_autos.arp). Here, more than 99.5% of the variation resided within subpopulations, regardless of the grouping.

In case of mitochondrial and Y-chromosomal data, the clusterings maximizing *F*_*CT*_ and minimizing *F*_*SC*_ values mirrored the patterns suggested by *Φ*_ST_ values (haplotypic AMOVA). The mtDNA distances indicate a grouping placing the Jívaro and the Pomacochas together and the remaining Chachapoya subsets in a different group, with the exception of Rodríguez de Mendoza which remains separate like the Huancas and the Cajamarca. For this scheme, 7.21% of the total variation resides between groups (*p* < 0.001) and 1.68% among populations within groups (*p* < 0.05) (S13 Table, run: 13_mito.arp). Similarly, for Y-chromosome data, the genetic distances suggested grouping only the Chachapoya subsets, Chillao, Chachapoya, Rodríguez de Mendoza and Leymebamba but keeping the rest of the datasets separate. Following this, the AMOVA run 13_Y.arp, gave the best results for *F*_*CT*_ (0.104, *p* < 0.003) and *F*_*SC*_ (0.016, *p* > 0.05) values (S13 Table).

## Discussion

The Chachapoya peoples flourished in the northeastern Peruvian montane forests until their encounter with the Inca and Spanish led gradually to the dissolution of the culture. Here we have assessed past population signals of the Chachapoyas persisting in the modern Peruvian genetic diversity. Despite cultural diversity among the Chachapoyas, we could not find clear signs of genetic substructure in this region. However, the high native genetic diversity observed sheds light on the complex, gender and region-specific population history of the Chachapoyas. Obviously, these inferences are to some extent limited by the contemporary data used, as the modern diversity has been shaped by the entire history of the target populations. Ancient DNA data could provide additional information, but only if sample sets representative in space and time could be obtained. Furthermore, one could also argue that the autosomal markers used in the ForenSeq kit, geared for forensic purposes, are not ideal for detecting substructure. This could stem from the relatively limited number of loci as well as from ascertainment bias, selective inclusion of loci in the forensics-aimed kits that maximize the diversity and power for identification of individuals (globally). Larger, genome-wide data sets would obviously offer better resolution for substructure detection, but the impact of the latter, ascertainment bias, is difficult to quantify for our data. Previous studies [49] have shown that 13 STR loci can detect substructure at population level, but due to the ascertainment bias, the distances are deflated, i.e. that the forensic markers are indeed less sensitive in detecting the population substructure. Similarly, as among the Chachapoyan subgroups no significant substructure was observed for more sensitive Y-chromosomal and mitochondrial data, the impact of the ascertainment bias is most likely of quantitative nature.

### Lack of genetic substructure within Chachapoyas

It is becoming increasingly clear through recent ethnohistorical and archaeological research that the Chachapoyas region has hosted considerable social, political, and cultural diversity before the Late Horizon or Inca period (1475–1532 CE) [6, 50]. Within Chachapoyas, places separated by few dozens of kilometers like Luya, Chilchos, and Rodríguez de Mendoza, have held major cultural differences e.g. in mortuary practices, pottery styles and agricultural systems [51–56].

Despite these cultural clues from the past, signs of genetic subdivision in the region are weak. Although genetic distances and allele (haplotype) frequencies point to genetic differentiation of some Chachapoya subgroups, Y-chromosomal, mitochondrial and autosomal markers all convey conflicting pictures, thus evincing for rather insubstantial substructure (HWE, genetic distances, cluster analyses and AMOVA).

Only one of the seven subgroups, Pomacochas, differed significantly from most others in both mitochondrial and Y-chromosome data (*Φ*_ST_ > 0.070). On the other hand, admixture analyses based on autosomal markers showed again no detectable population substructure within Chachapoyas. Here, regardless of the hierarchical setting (intercontinental, intracontinental and local), the Chachapoya samples clustered together (Fig 3 and S5 Fig). However, despite the lack of significant substructure within the Chachapoyas region, a few statistically significant differences of mitochondrial haplogroup distributions were observed, e.g. lack of haplogroup A2 in Rodriguez de Mendoza. All these observations suggest that there are true differences in the frequencies of some haplogroups and certain degree of differentiation at some localities which echoes in the cultural diversity discussed above. Recent gene flow obviously may have obscured the past differentiation. Yet, ancient substructure is known to leave persistent demographic signals especially in the haploid markers, as shown e.g. in Finland [57].

#### DNA suggests Pomacochas—Jívaro connections

Instead of substructure and disconnection, mitochondrial and autosomal data shows that the northern section of the Chachapoyas region, the Pomacochas area, share genetic affinity with Jivaroan populations.

There is lack of archaeological research on whether the territory around Pomacochas and further north (Jumbilla and Yambrasbamba) was part of the Chachapoyas realm but potential historical connections between these two areas cannot be completely ruled out. It has been suggested that the historical Jívaro and the ancient Chachapoya peoples shared some cultural features [55, 58], however, the available archaeological evidence is yet insufficient to sustain this assumption. Despite this, the Chachapoyas region has been identified as a crossroads, at the center of interregional exchange networks throughout millennia [59]. Indeed, even early lithic assemblages found in the Chachapoyas region resemble lithic industries from as far as the north Peruvian coast (Paiján) and highland Ecuador (El Inga) [59] which account for the uniqueness of this region. From the genetics point of view, two recent independent studies [60, 61] have identified east-west gene flow linking the north Peruvian coast and highlands with Amazonian groups to the east of the northern Andes. Interestingly, one of these studies has found gene flow between the Chachapoyas and populations from the Jivaroan ethnolinguistic stock such as the Awajún and Candoshi [61]. This evidence gives further support to our finding despite the lack of published historical works on ancient or recent migrations from Jivaroan populations towards the Chachapoya territory or vice versa. As such, this finding provides new ground for archaeological and ethnohistorical research investigating cultural interactions between these areas.

#### Chachapoyas harbour an autosomal variation component of undetermined origin

Among the Chachapoyas and the Jívaro, admixture analyses revealed- in addition to Native American and Caucasian components—a third autosomal component that could not be readily associated to any modern source population. Within Chachapoyas, inter-subgroup differences in the commonness of this component was observed especially in Rodríguez de Mendoza, but nearly absent in e.g. Chillao, La Jalca and Uchucmarca. On the regional level, the component was only present among the Chachapoyas and the Jivaroan Awajún and Wampís. In order to understand the origin of this variation, individuals harboring ≥ 60% of this third component were regrouped and genetic distances between this group and reference populations from the Americas were estimated. These comparisons, however, were unable to shed light on the origin: the synthetic “3^rd^ component group” showed no obvious affinity to reference datasets of African, European or Asian origin, nor to the populations included from the Americas. In analyses with more reference populations but less data (10 aSTRs), the group remained distant to all the other populations except the Amazonian Cashibo from Peru. The exact origin of this variation component remains thus elusive; it could derive from a yet unsampled ancient or modern population, for example representing assemblages that have developed locally in semi-isolated populations with no distinctive cultural features. On this note, a recent study [62] of South American populations has shown the existence of two autosomal components of alleged Amazonian origin: 1) The first one, distributed along the eastern slopes of the Northern Andes in Ecuador and Colombia, and 2) the second one, observed mostly in populations from the Peruvian rainforest. Due to geographic proximity and the commonness of the 3^rd^ unknown component among the Amazonian Jívaro, we could suggest ties to the second component, although this cannot be tested with our current resolution.

### Asymmetrical European gene flow in the Chachapoyas region

As in several previous studies on South American populations [63, 64], we observed gender-biased European gene flow into Chachapoyas: 5% of the mitochondrial and 42% of the Y-chromosomal haplotypes belonged to haplogroups typical for Europeans (such as H and U in mitochondrial; R and I in Y-chromosome). A substantial proportion of non-Native American Y-chromosomal lineages were also observed in Cajamarca, but this was not the case in Jívaro and Huancas.

During the conquest, the Spanish formed close alliances with the Chachapoyas against the Inca, and established the city of Chachapoyas as capital of the region. As the center of Spanish rule, and currently the largest town in the region, the Chachapoya subgroup (which includes also samples from Levanto) would be expected to host a higher proportion of European alleles but the highest European contribution (autosomes > 50%) was found instead in Kuelap, Leymebamba, and Rodríguez de Mendoza. In fact, the proportion of non-Native American haplotypes peaked in Leymebamba and Rodríquez de Mendoza (≥ 58%). Of these three areas, high European contribution in Leymebamba could be linked to its role as a gatekeeper to the Chachapoyas region, since it is one of the most important settlements on the eastern side of the mountains along the only road in the region that crosses the Andes and the Marañon River. This road system connected the Chachapoya and the Cajamarca regions, as well as Uchumarca in the south, already before the Spanish conquest [4], which may also have facilitated non-Native American gene flow during the Early Colonial Period. In the area around Rodríguez de Mendoza, Spanish settlements were already observed during a visit of archbishop Mogrovejo to the area in 1593 [65]. Thus, this area became a preferred destination for the permanence of European settlers echoing also in the genetic makeup of local residents. This was probably the result of a combination of factors, such as warmer climate, fertile soil and large rivers. In addition, its location, closer to the lush Amazonian rainforests, may have been of interest for Spanish conquistadors when pursuing expeditions in search of El Dorado [4, 10] which may have prompted the settlement of more Spanish people in the area. The high European genetic contribution observed in the Kuelap group is, in turn, slightly puzzling as the Inca and Spanish cultural presence on the left bank of the Utcubamba river, where this sample set comes from, seems to have been less significant than on the other side [4, 51, 59, 66]. Interestingly, the Chillaos subgroup harbors less European admixture, complying somehow with this pattern described by archaeology and ethnohistory. Although Kuelap was abandoned shortly after the Spanish incursions in this area, the site is recognized as one of the most important archeological settlements within the Chachapoyas region and its significance as a center of religious and political power for the local populations [67, 68] may have transcended far beyond the Inca times. However, the site is seldom cited in early documents and was rediscovered late during the 19^th^ century [6, 67]. The mismatches observed between the genetic and cultural evidence point to increasingly complex patterns of European interaction with the local populations at even smaller scales.

### Demographic trajectories

For mitochondrial DNA, coalescent-based BSPs are extensively used methods to make inferences of the changes in the past female population size [69]. However, since for Y-STRs data similar approaches are not equally well established, past male population dynamics are not routinely assessed in population genetic studies. A comprehensive estimation of the male effective population sizes would require Y-chromosomal sequence data which is seldom produced. Nevertheless, to be able to compare the signals in the male and female population histories, we assumed an experimental approach to construct BSPs with Y-STR markers. The temporal congruence in the Y-chromosome and mtDNA-based BSP patterns gives credence for the validity of the Y-chromosomal results obtained.

Thus, demographic histories in northeast Peru were explored with BSPs, using both mitochondrial sequences and Y-chromosomal STR haplotypes among the study populations. While the effective population sizes reconstructed by the BSPs are largely conjectural, the overall trends are revealing. The BSP patterns obtained from our data differed both, between sexes (markers), and regions (Andean/Amazonian).

In general, all BSP plots showed an ancient population expansion period that began at 625–500 generations ago i.e. 15.6–12.5 kya (a 25-year generation interval is assumed for all datings, unless indicated otherwise), a range that encompasses the earliest evidence of human presence in the continent [70, 71]. The connection of this early expansion to any real population events other than the entry in the Americas is nebulous, but the later developments are intriguing: the graphs show no signs for a mid-Holocene demographic crisis evident in South American archaeological radiocarbon data and palaeoclimatic evidence [72]. Instead, the Y-chromosomal and mitochondrial data demonstrate consistent patterns of population decline later in time, starting at around 125 generations ago i.e. 3.1 kya. This decline appears to temporally roughly coincide with a period of decreased solar irradiation (“The Homeric minimum”) which has been associated to global climatic change, including heightened El Niño oscillation [73, 74], and to major cultural shifts in Europe [75]. Interestingly, pollen and charcoal records from sediments of Lake Pomacochas in the Chachapoyas region demonstrate substantial changes in landscape around 3 kya, pointing somewhat paradoxically to heightened human activity. Increase on charcoal and *Poaceae* pollen, decrease of arboreal pollen and appearance of maize in the sedimentary record around this time all point to the clearing of forests for maize cultivation [76].

#### Sex-specific differences

The effective population size trajectories differ between sexes for the last 125 generations i.e. 3 kya. In the recent past, BSPs based on mitochondrial sequences show a steep but a relatively continuous decline which becomes severe over time but does not show a clear low point of population contraction. In contrast, the Y-chromosome BSP for the Chachapoyas sample set registers a steep population decline that reaches the lowest point c. 25 generations ago. This bottleneck would match the Inca or the Spanish conquest if a generation interval of 23 or 20 years, respectively, is assumed, which seem at least marginally realistic. These generation interval estimates would date the start of the general decline at 125 generations ago (above) to c. 2 850 and 2 500 BP.

The BSPs also suggest that the Chachapoya male population was nearly halved sometime between the 15^th^-17^th^ centuries. It is tempting to associate demographic events as drastic as this with the Inca and Spanish conquests. During the Late Horizon (1475–1532 CE), the relocation policies applied by the Incas are recognized as important contributing factors to the depopulation in the Chachapoya region but these are not described as severe as the demographic collapse during and after European contact (multiple infectious diseases and pandemics over a prolonged time, war, selective relocation policies and forced labor) [11, 77]. However, all these events should have affected both males and females with equal acuteness, which is in contrast with the results. In addition to the above-mentioned evidence for general demographic collapse, males especially were affected by the Inca and Spanish military conscriptions. For instance, Chachapoyan males were recruited to fight the Inca as well as to participate in the early colonial El Dorado expeditions. Several of these expeditions to the tropical Amazonian rainforests started in towns within the Chachapoya region [10]. This would suggest that the severe male-biased population contraction may be associated with post-European contact events.

#### Regional patterns

In addition to the sex-specific differences, there are notable dissimilarities in the shape of BSPs between Andean and Amazonian reference populations. In general, the Andean male and female populations show higher effective population sizes for the last 250 generations, and clear signs of population growth in the past. The Amazonian populations exhibit a starkly contrasting pattern with smaller overall sizes and very moderate or non-existent population growth. This contrasting Andes/Amazon demographic pattern could reflect different levels of isolation and resource availability. This kind of regional dynamics has earlier been suggested for South America, based on Y-chromosomal data [78], and has also been proposed to explain global linguistic diversity [79]. Within Chachapoyas, most of the subgroups show Andean-type demographic trajectories, except Pomacochas and Corobamba, with patterns resembling those of the Amazonian populations. This adds to the earlier evidence of Amazonian (Jivaroan) affinity of the Pomacochas subgroup.

The growing effective population sizes in the Andean populations and in the Chachapoyas, as well as the overall high diversity in the Chachapoyas region, differ markedly from other South American Native populations. Interestingly, *in silico* analyses of species richness in South America from Late Quaternary until present have identified this region in northeastern Peru and southwestern Ecuador as one of the hotspots of biodiversity, also confirmed by empirical observations [80]. Here, birth and persistence of biological diversity in this region is most likely driven by topography and climate [80]. These rich ecosystems then provide more resources for the local human communities and could have contributed to the observed genetic diversity by allowing larger and more stable population sizes through time [81–83]. This area between the Amazonian rainforest and Andean mountains hosts different kind of ecosystems over a relatively short geographic distance, which may have buffered the local populations—human and non-human—in times of environmental change [84].

## Concluding remarks

To the extent of our statistical resolution, we did not detect population substructure in the Chachapoyas sample, which accounts for the notion that genetic subdivision does not necessarily accompany cultural differentiation. In other regions of South America, population substructure has also been found to be negligible in groups from the same and different language stock [85–87] In line with previous research [1, 2], we found exceptionally high levels of local genetic diversity in the Chachapoyas for both lineage markers that together with the large effective population sizes indicate particular demographic processes in this region driven most likely by topography and climate. This is also consistent with previous studies suggesting similar areas, at the junction of montane and tropical rainforests along the eastern slopes of the northern Andes, are as well identified as hotspots of human genetic diversity [86, 88–91]. In addition to these findings, we detected gene flow between the Amazonian Jívaro and an area situated at the north of the Chachapoyas territory here represented by the Pomacochas subgroup (which included also the localities of Jumbilla and Yambrasbamba). Gene flow events between neighboring territories such as these are not surprising as long-distance genetic exchanges of varying magnitude at this latitude have been observed between the Andean and Amazonian domains [91–93], and even with the Pacific Coast [60, 94]. Additionally, an unknown autosomal component has been identified among the Chachapoyas and the Awajún and Wampís. Although the origin of this component could not be clarified, we speculate it may be of Native American origin as it remains distant to African, European and Asian populations.

European admixture impacted the indigenous genetic variation at various scales, from macro-regions (e.g. Amazon, Andes) to populations [95, 96]. In this study, both lineage and autosomal markers point to asymmetrical (mtDNA and Y-chromosome) European gene flow in the Chachapoya territory. For autosomal data these signatures of European admixture were heightened in the subgroups Kuelap, Leymebamba and Rodríguez de Mendoza. The geographic position and cultural importance of these areas are highlighted as explanations to these patterns, showing that differences can be detected even at smaller spatial scales. As the dates of admixture between the European and Native American components cannot be estimated from the current data, we should consider also the possibility that more recent admixture events may have concealed the patterns established during the Early Colonial Period.

The female demographic signature of the Chachapoyas as a whole and the Jivaroan set resemble the one from the Andean area whereas the male population history in the Chachapoyas is more similar to the Amazonian one. Larger long-term effective population sizes have been previously characterized for the populations from the Andean area which contrasts with other regions of South America [78, 97]. Moreover, comparisons of both male and female demographic histories also indicate a more severe population decline on males which finds support in the historical portrait of the northeast Peruvian Andes as an area that experienced a dramatic population decline, particularly among males [4]. Even within the Amazonian region, genome wide studies in South America have shown that population sizes have been shaped by different demographic processes throughout their history [62, 92]. In that sense, in addition to the detection of genetic drift by means of autosomal data, it is also vital to examine demographic histories based on lineage markers, as different processes affecting the male and female population sizes can result in divergent patterns like the ones observed in this and other studies e.g. [98]. We acknowledge that although various inferences about the population structure and population history in the Chachapoyas have been provided here, other questions such as the origin of the Chachapoya ancestry can ultimately be addressed with higher resolution and/or ancient DNA data.

## Supporting information

S1 Fig Permutated haplogroup distributions and actual observations showing the deviations (*p* < 0.05) with arrows in Chillao, Pomacochas and Jívaro. (TIF)

S2 Fig Mitochondrial MDS plot with populations from the Americas showing the Pomacochas and the Rodríguez de Mendoza subgroups as well as the pooled remaining subgroups under the label Chachapoyas. (TIF)

S3 Fig Mitochondrial coding region BSPs for all Chachapoyan subgroups. (TIF)

S4 Fig Y-chromosome MDS plot with populations from the Americas showing the Chachapoyan subgroups pooled with exception of La Jalca and Pomacochas. (TIF)

S5 Fig Admixture plot including study and reference populations for K = 4. (TIF)

S6 Fig In the Chachapoyas set, a value of K = 3 was consistently estimated by two methods, ln *P* (*X*|*K*) and Δ*K*. (TIF)

S7 Fig PCoA plot showing the study populations and the set CAU (US Americans with European ancestry). The circle indicates Individuals harboring > 60% of the “3^rd^ unknown component”.
(TIF)

S8 Fig MDS plots of autosomal genetic distances showing the individuals in the “3^rd^ unknown component”. Study populations and Chachapoyan subgroups in black, m: mestizo. (A) 116 loci. (B) 10 loci.
(TIF)

S1 Table Multiplexes M3 and M346. (A) Genotyping. (B) Primer information grouped by multiplexes.
(XLSX)

S2 Table Sample sizes and reference populations. (A) mitochondrial DNA. (B) Y-Chromosome. (C) autosomal data.
(XLSX)

S3 Table Sample sizes and best substitution models for mitochondrial DNA data BSPs. (XLSX)

S4 Table Sample sizes, supported clock model and prior parameters for Y-chromosome data BSPs. (XLSX)

S5 Table Absolute frequency of mitochondrial sub-haplogroups in all study populations. Frequencies only from our datasets.
(XLSX)

S6 Table Diversity indices for mitochondrial DNA data. (A) Non-Native American haplotypes are included in the study populations. (B) Non-Native American haplotypes are excluded in the study populations.
(XLSX)

S7 Table Mitochondrial *Φ*_ST_ distances. (A) Non-Native American haplotypes are included in the study populations. (B) Non-Native American haplotypes are excluded in the study populations.
(XLSX)

S8 Table Mitochondrial *Φ*_ST_ distances when the Chachapoyan subgroups are pooled. Non-Native American haplotypes are excluded in the study populations.
(XLSX)

S9 Table Y-chromosome haplotypes (Guevara et al. 2016) and SNP assignment by multiplexes (This study). (XLSX)

S10 Table Absolute frequency of Y-chromosome sub-haplogroups in all study populations. Frequencies only from our datasets.
(XLSX)

S11 Table Diversity indices. (A) 23 Y-STRs. (B) 17 Y-STRs.
(XLSX)

S12 Table Y-chromosome *Φ*_ST_ distances. (A) 23 Y-STRs when the Chachapoyan subgroups are not pooled. (B) 17 Y-STRs when the Chachapoyan subgroups are pooled.
(XLSX)

S13 Table AMOVA results for different kind of marker systems. (XLSX)

S1 File STRUCTURE plots including study populations and datasets from different continental origins (K = 2–6). (PDF)
