# Supplementary figures and images for: Genetic assessment reveals no population substructure and divergent regional and sex-specific histories in the Chachapoyas from northeast Peru
Source: PLoS One. 2020 Dec 31;15(12):e0244497. doi: 10.1371/journal.pone.0244497 (PMC7774974; doi:10.1371/journal.pone.0244497)

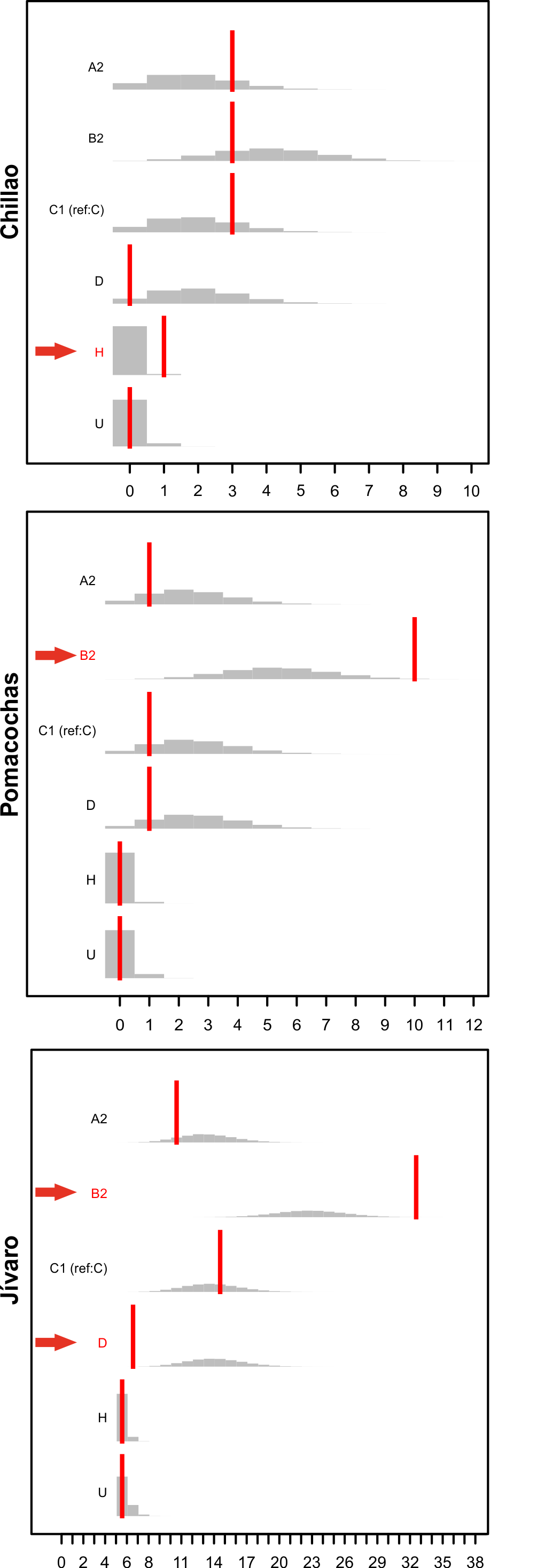

Supplement: S1 Fig — (TIF) [file pone.0244497.s001.tif]

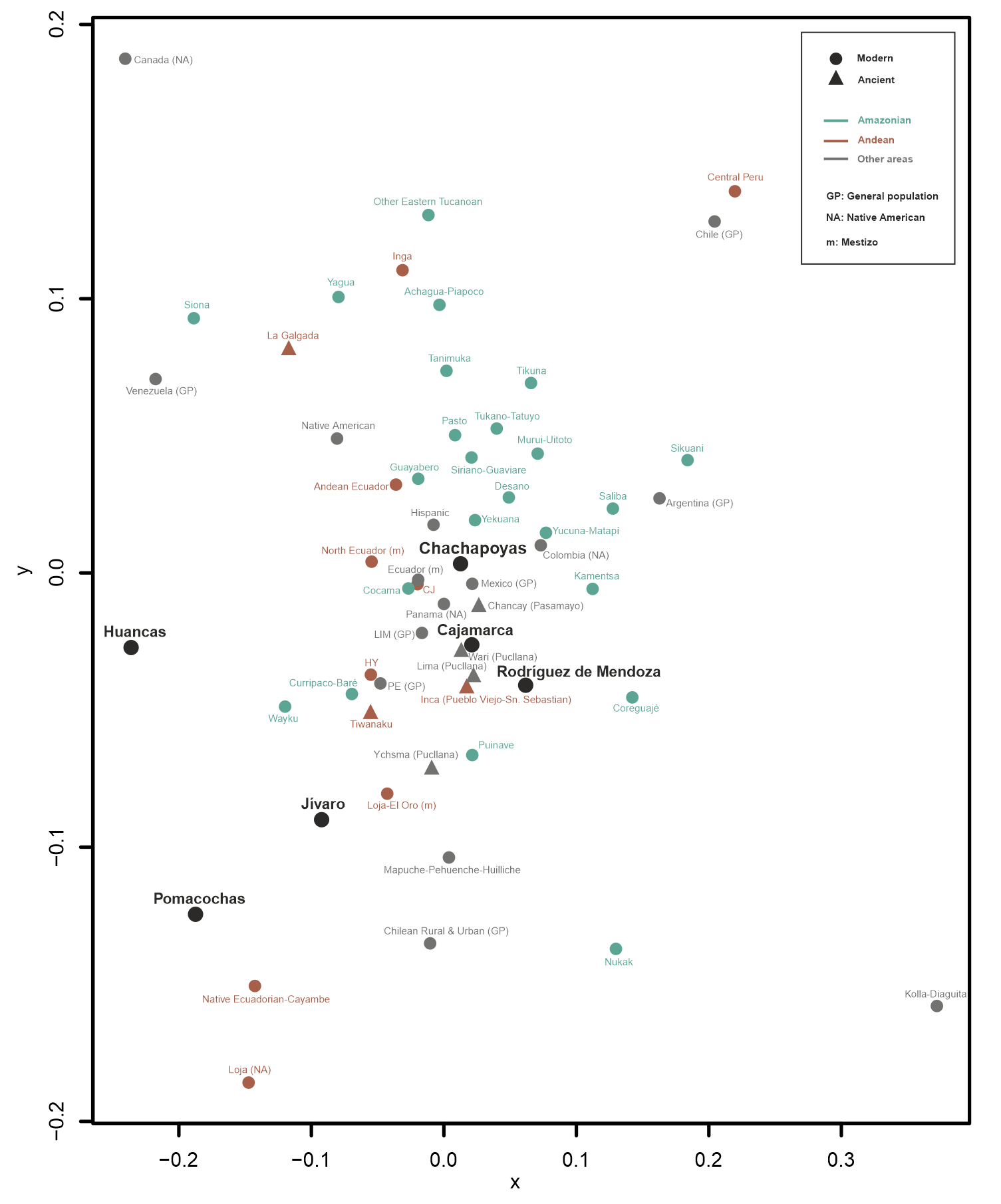

Supplement: S2 Fig — (TIF) [file pone.0244497.s002.tif]

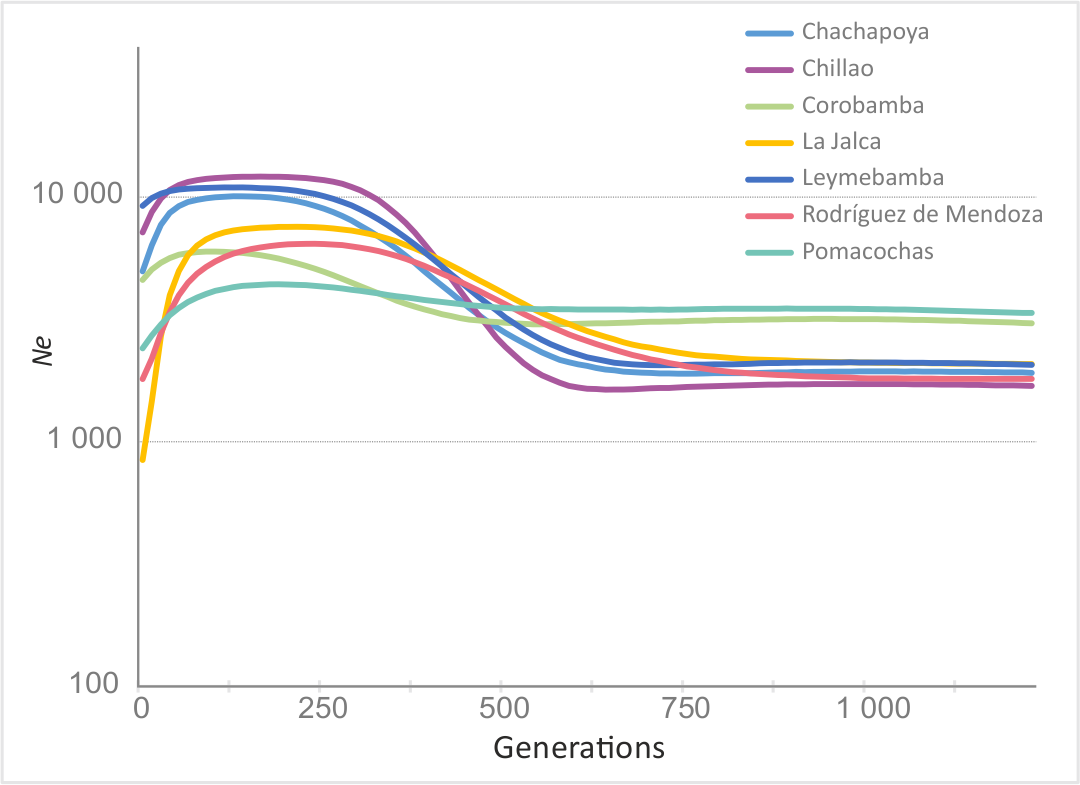

Supplement: S3 Fig — (TIF) [file pone.0244497.s003.tif]

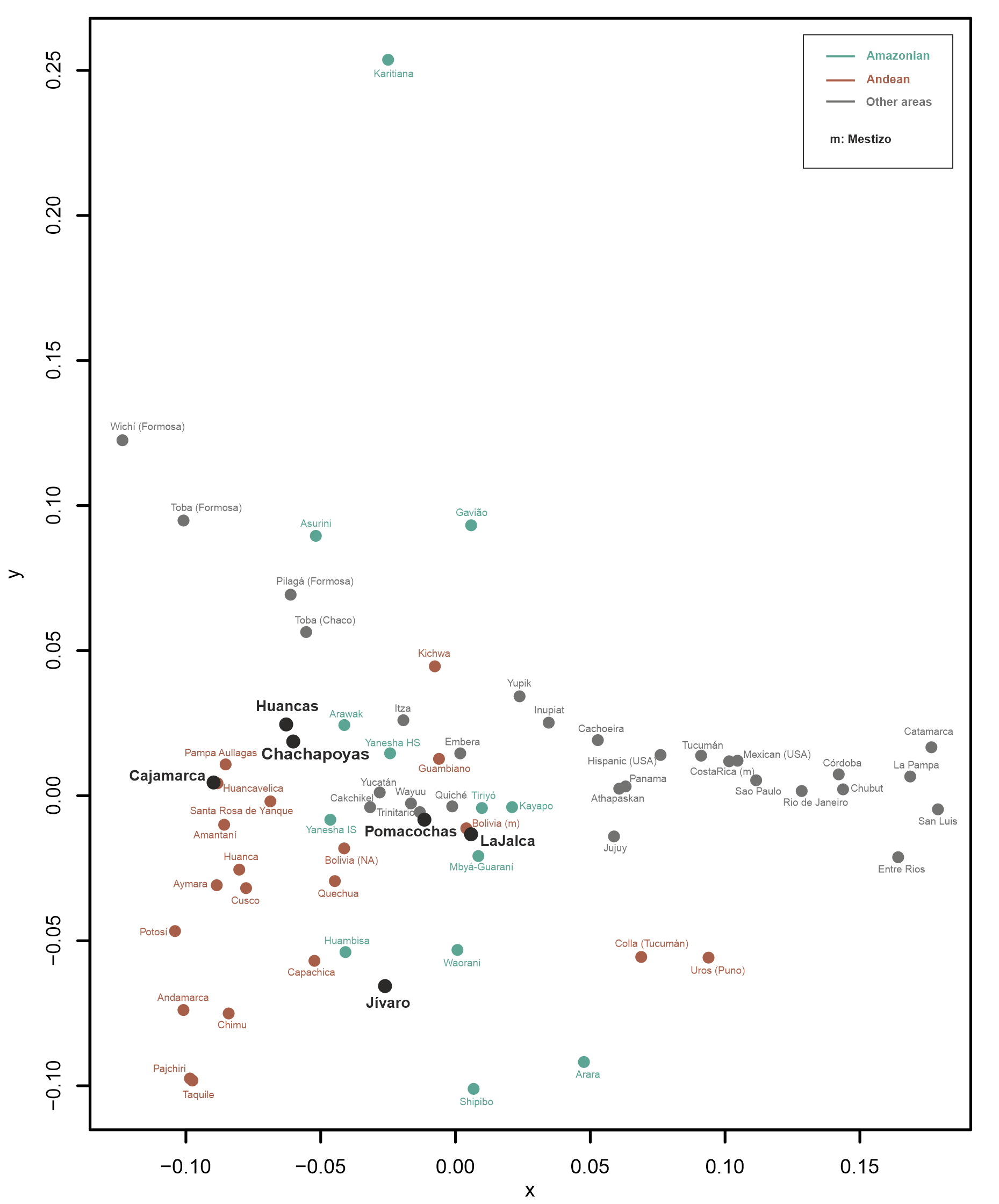

Supplement: S4 Fig — (TIF) [file pone.0244497.s004.tif]

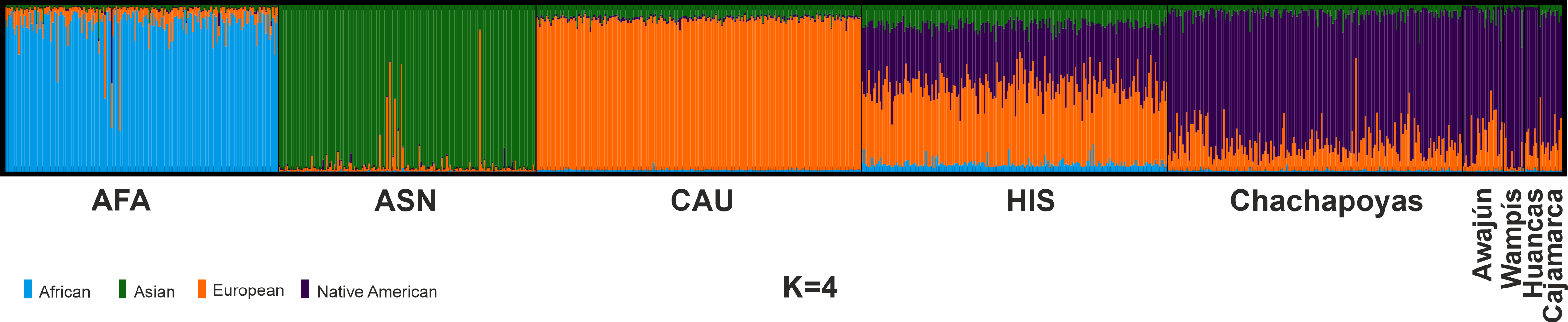

Supplement: S5 Fig — (TIF) [file pone.0244497.s005.tif]

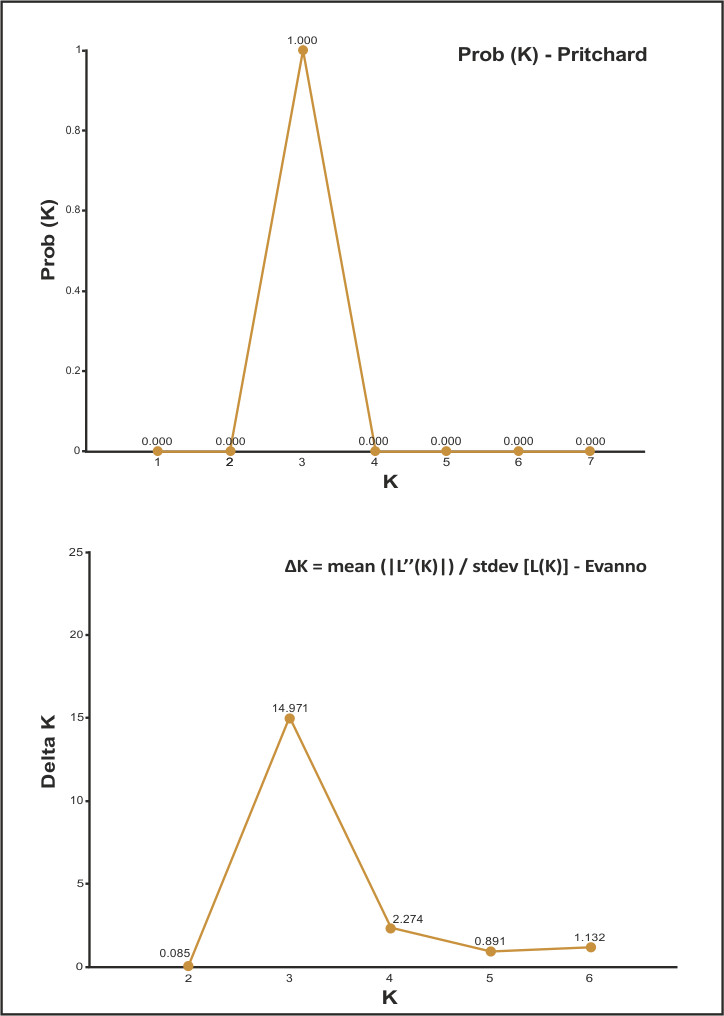

Supplement: S6 Fig — (TIF) [file pone.0244497.s006.tif]

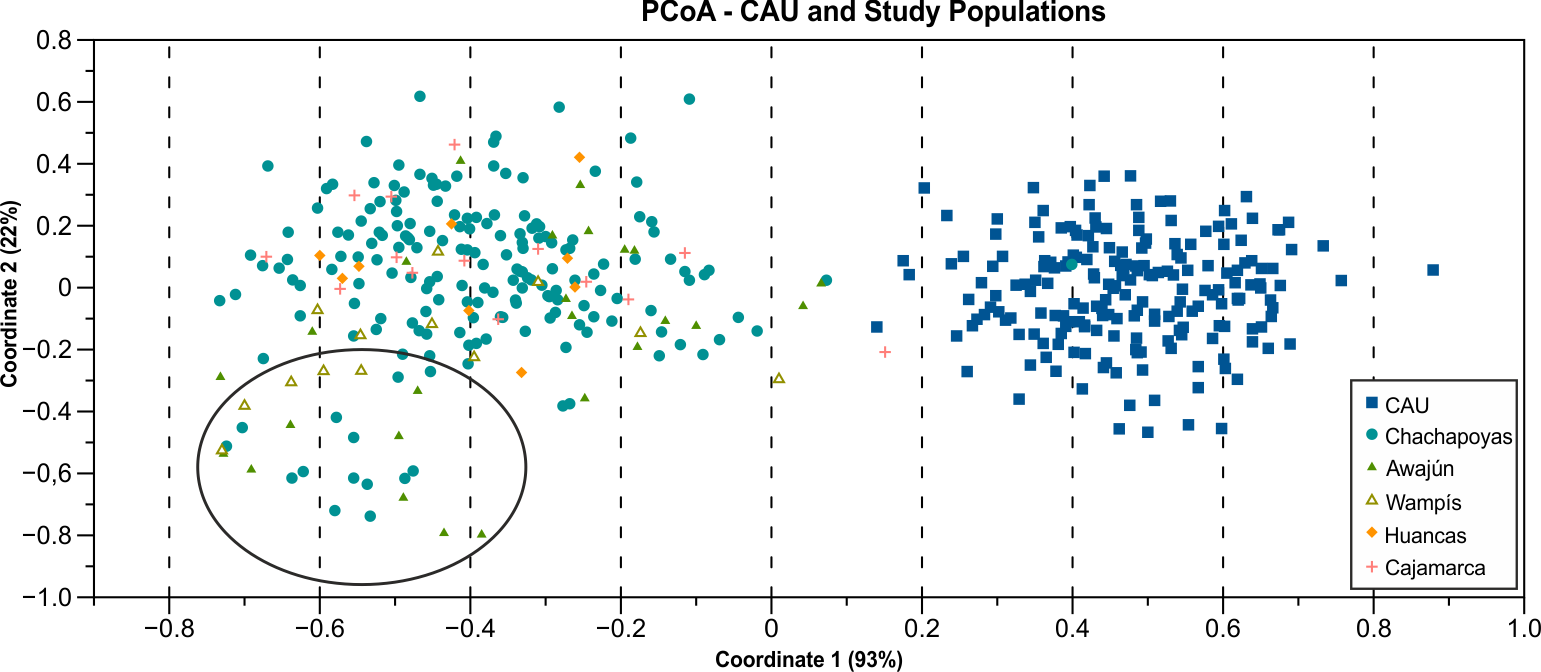

Supplement: S7 Fig — The circle indicates Individuals harboring > 60% of the “3rd unknown component”. (TIF) [file pone.0244497.s007.tif]

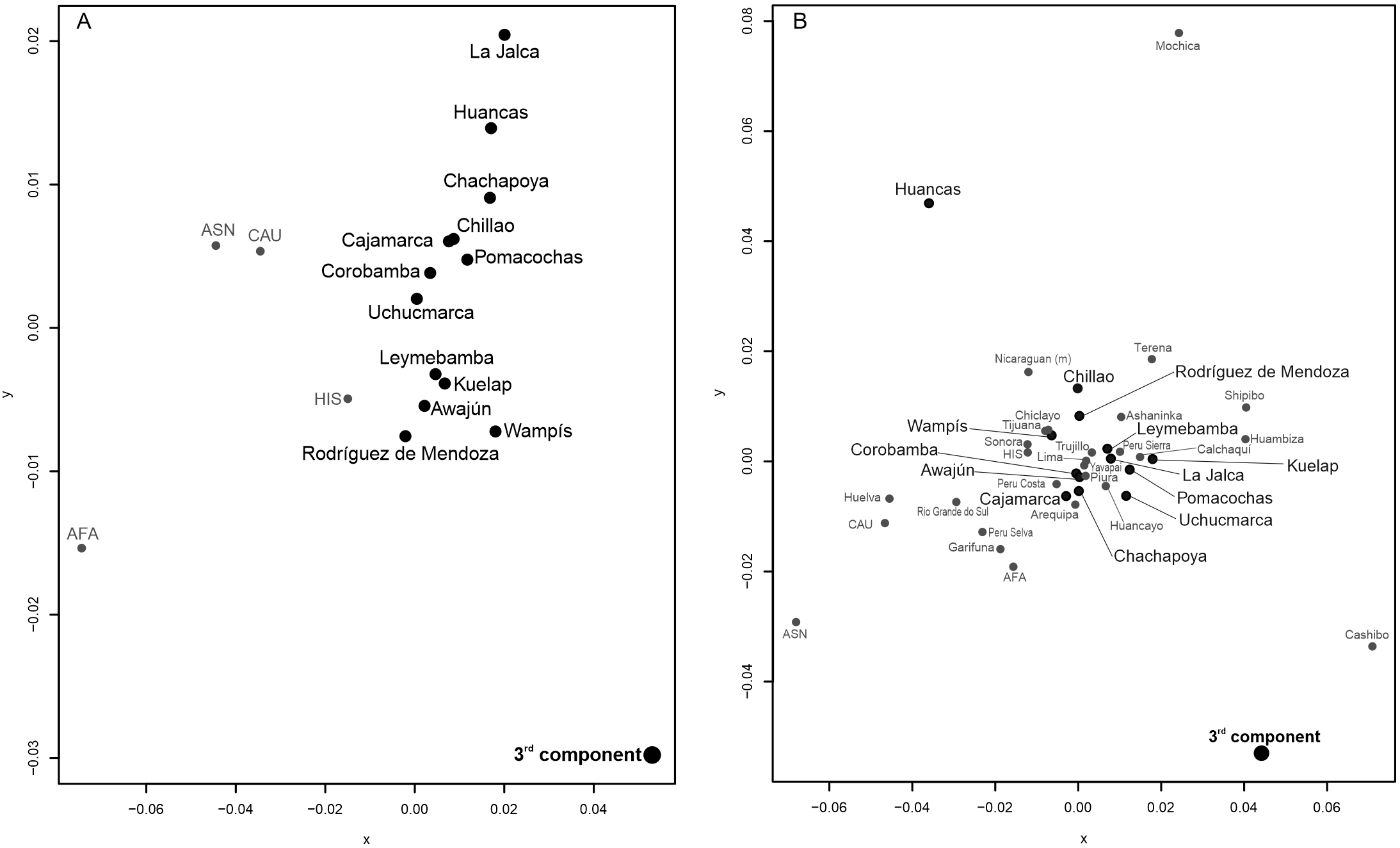

Supplement: S8 Fig — Study populations and Chachapoyan subgroups in black, m: mestizo. (A) 116 loci. (B) 10 loci. (TIF) [file pone.0244497.s008.tif]
