# Supplementary material for: Genetic assessment reveals no population substructure and divergent regional and sex-specific histories in the Chachapoyas from northeast Peru
Source: PLoS One. 2020 Dec 31;15(12):e0244497. doi: 10.1371/journal.pone.0244497 (PMC7774974; doi:10.1371/journal.pone.0244497)

Study populations and European (CAU)

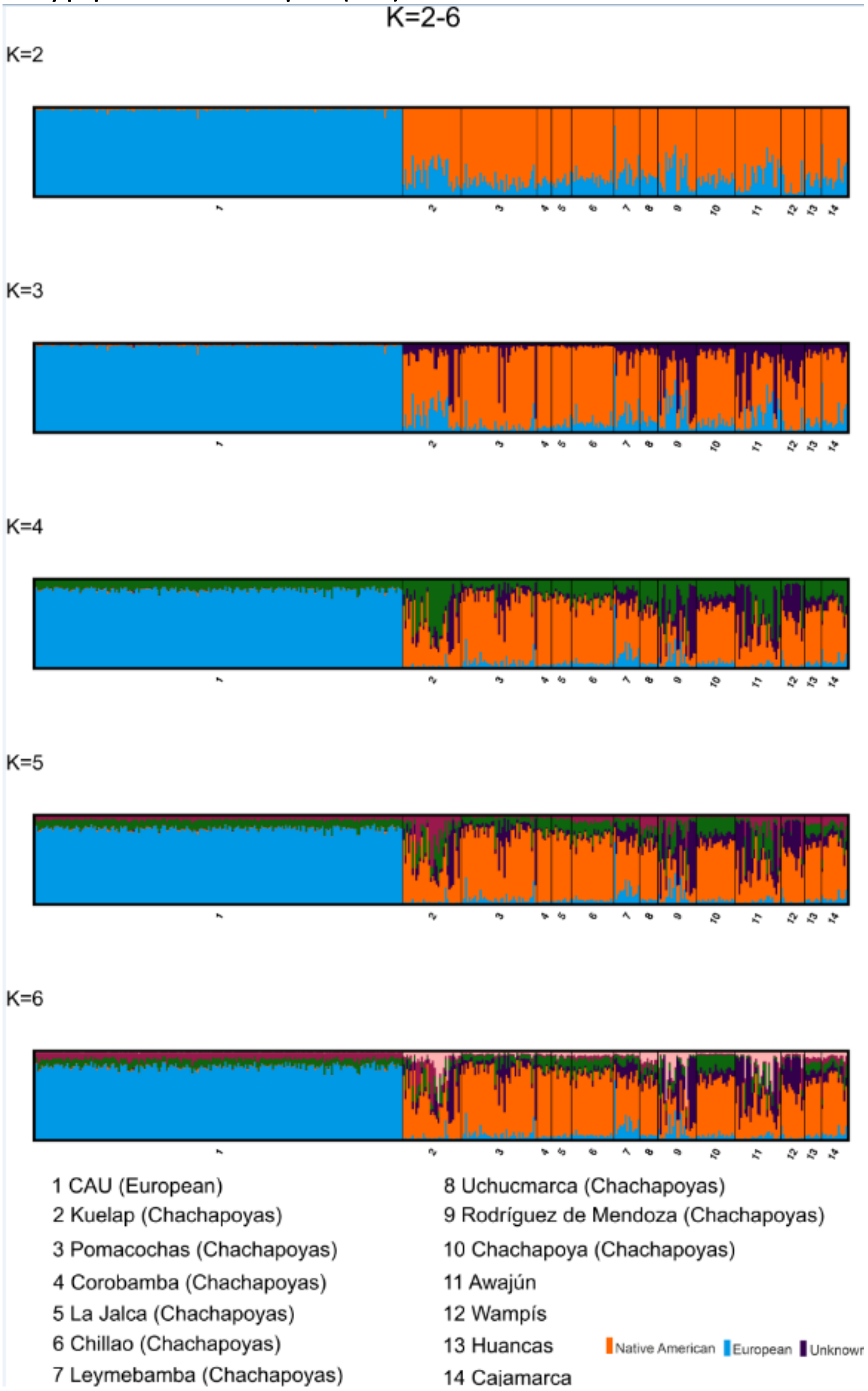

Study populations and Asian (ASN)

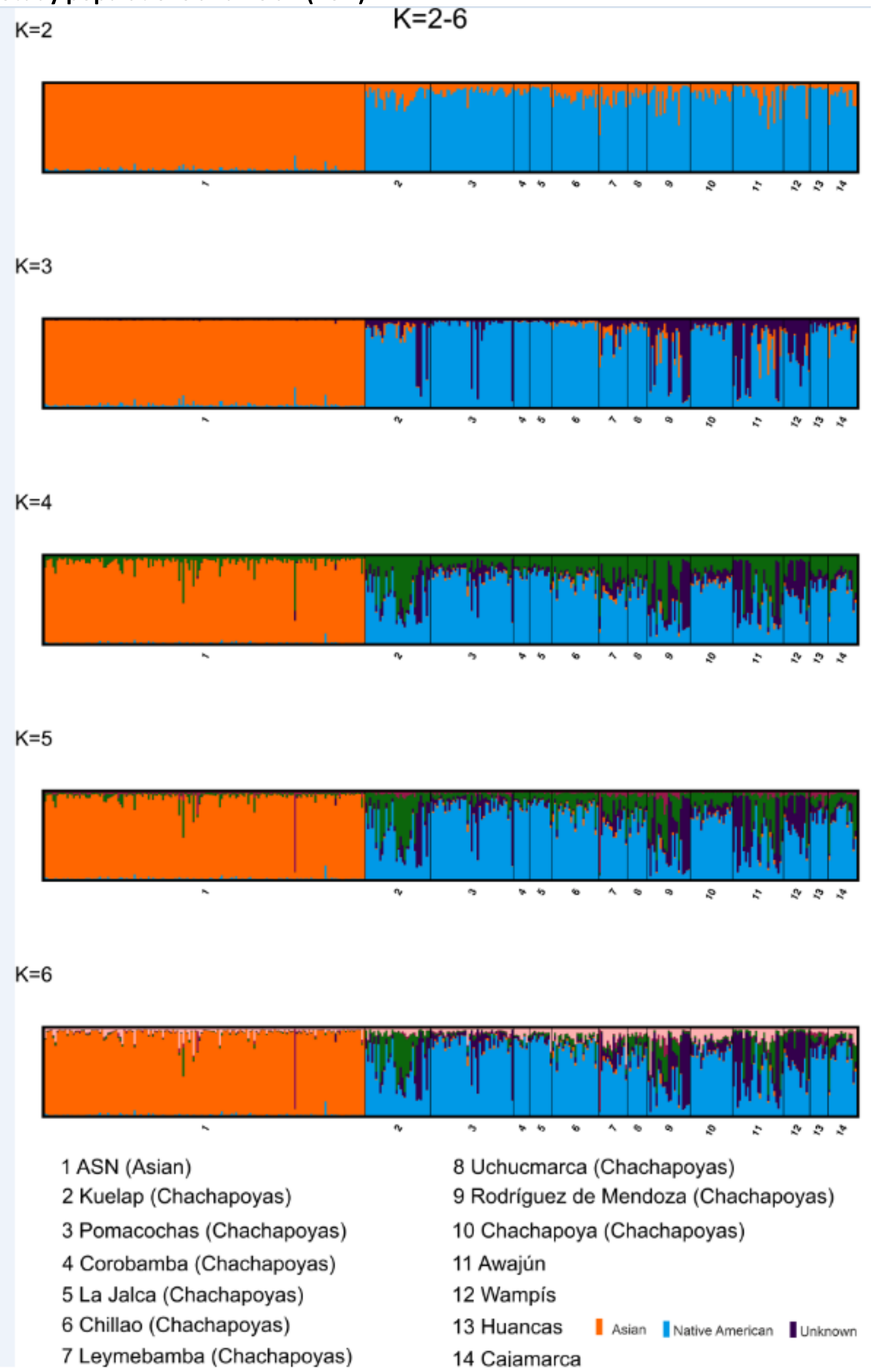

Study populations and Hispanic (HIS)

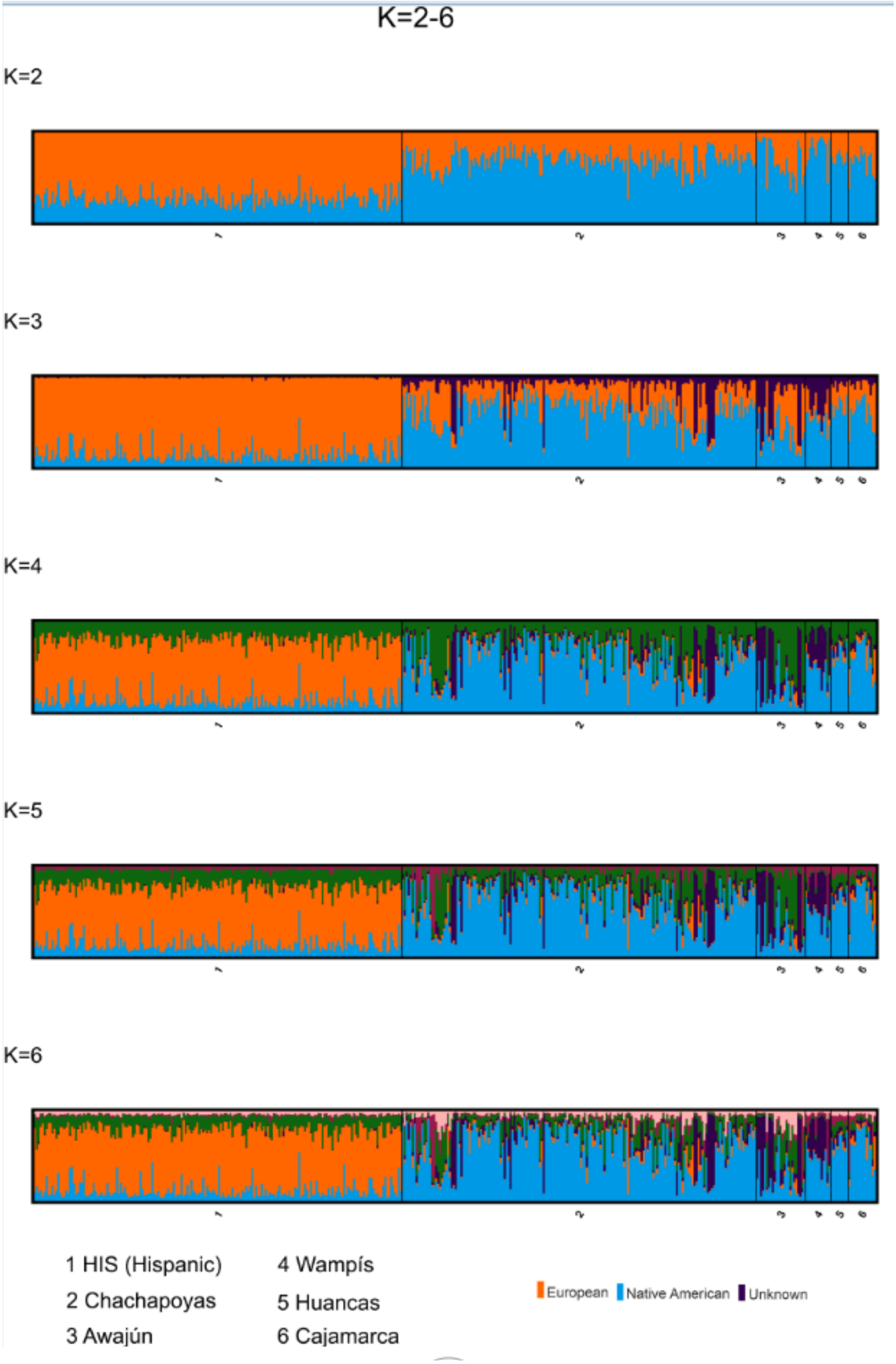

Supplement: S1 File — (PDF) [file pone.0244497.s022.pdf]
